# Reviewer acknowledgement 2013

**DOI:** 10.1186/1757-1146-7-6

**Published:** 2014-01-31

**Authors:** Hylton Menz, Mike Potter

**Affiliations:** 1 La Trobe University, Bundoora, Australia; 2 University of Southampton, Southampton, UK

## Abstract

**Contributing reviewers:**

The Editors of *Journal of Foot and Ankle Research* would like to thank all our reviewers who have contributed to the journal in Volume 6 (2013).

## 

Michael Backhouse

United Kingdom

Richard Baker

United Kingdom

Ruth Barn

United Kingdom

Christian Barton

Australia

Shan Bergin

Australia

Ivan Birch

United Kingdom

Alison Blake

United Kingdom

Daniel Bonanno

Australia

Catherine Bowen

United Kingdom

Frank Bowling

United Kingdom

Helen Branthwaite

United Kingdom

Ivan Bristow

United Kingdom

Gloria Bueno

Spain

Joshua Burns

Australia

Paul Butterworth

Australia

Jackie Campbell

United Kingdom

Matt Carroll

New Zealand

Sanghoon Cha

Korea South

Nicole Chimera

United States of America

Vivienne Chuter

Australia

Matthew Cichero

United Kingdom

Matthew Cotchett

Australia

Sarah Curran

United Kingdom

Hamish Curry

Australia

Beverley Durrant

United Kingdom

Mairghread Ellis

United Kingdom

Angela Evans

Australia

Lisa Farndon

United Kingdom

Jill Ferrari

United Kingdom

Aharon Finestone

Israel

Daniel Fong

China

Charles Fox

United Kingdom

Alfred Gatt

Malta

Kathleen Gatt

Malta

Paul Gibbons

Australia

Nicholas Giovinco

United States of America

Ward Glasoe

United States of America

Ravindra Goonetilleke

Hong Kong

Taryn Gordon

United Kingdom

Andrea Graham

United Kingdom

Kelly Gray

Australia

David Gutekunst

United States of America

Jill Hall

United Kingdom

Jill Halstead

United Kingdom

Joseph Hamill

United States of America

Farina Hashmi

United Kingdom

Fiona Hawke

Australia

Tong-Chuan He

United States of America

Gordon Hendry

United Kingdom

Matthew Hoch

United States of America

Lindsey Hooper

United Kingdom

Matthias Hösl

Germany

Ru-Lan Hsieh

Taiwan

Richard Jones

United Kingdom

Anagha Joshi

United Kingdom

Tim Kilmartin

Ireland

Kevin Kirby

United States of America

Hirofumi Kosaka

American Samoa

Ozkan Kose

Turkey

Peter Lazzarini

Australia

Alberto Leardini

Italy

Kathy Liu

United States of America

Christopher Maclean

Canada

Maren Mahowald

United States of America

Ian Mathieson

United Kingdom

Joanne Mccardle

United Kingdom

Alistair Mcinnes

United Kingdom

Nichola Mclarnon

United Kingdom

Jasmine Menant

Australia

Karen Mickle

Australia

Stewart Morrison

United Kingdom

Shannon Munteanu

Australia

Pedro Munuera

Spain

George S Murley

Australia

Deborah Nawoczenski

United States of America

Christopher Nester

United Kingdom

Christopher Neville

United States of America

Sheree Nix

Australia

William Parr

Australia

Joanne S Paton

United Kingdom

Byron Perrin

Australia

Carlos Pique-Vidal

Spain

Julia Potter

United Kingdom

Stephen Preece

United Kingdom

Trevor D Prior

United Kingdom

Smita Rao

United States of America

Anita Raspovic

Australia

Michael Skovdal Rathleff

Denmark

Anthony Redmond

United Kingdom

Sarah Reel

United Kingdom

Diane Riddiford-Harland

Australia

Edward Roddy

United Kingdom

Keith Rome

New Zealand

Dieter Rosenbaum

Germany

Cristina Sartor

Brazil

Hans Savelberg

Netherlands

Rolf Scharfbillig

Australia

Michael Schwarze

Germany

Zhongmin Shi

China

Tzyy-Yuang Shiang

Taiwan

Simon Smith

Australia

Achini Soysa

Australia

Martin Spink

Australia

Kate Springett

United Kingdom

Michelle Spruce

United Kingdom

Julie Stebbins

United Kingdom

Martijn Steultjens

United Kingdom

Maria Stokes

United Kingdom

Minna Stolt

Finland

Yasuhito Tanaka

Japan

Scott Telfer

United Kingdom

Dominic Thewlis

Australia

Paul Douglas Tinley

Australia

Michael Tirgan

United States of America

Stewart Tomlinson

United Kingdom

David Torgerson

United Kingdom

Kirsten Tulchin-Francis

United States of America

Stephen Urry

Australia

Wesley Vernon

United Kingdom

Steven Walmsley

Australia

Stuart Warden

United States of America

Martin Warner

United Kingdom

Scott Wearing

Australia

Caleb Wegener

Australia

Wendi Weimar

United States of America

Kwi Hwan Whang

South Korea

Anita Williams

United Kingdom

Cylie Williams

Australia

Peter Worsley

United Kingdom

Cynthia Wright

United States of America

Yongping Zheng

Hong Kong

